# Serum Indoxyl Sulfate as a Potential Biomarker of Aortic Stiffness in Persons with Type 2 Diabetes Mellitus

**DOI:** 10.3390/medicina61091607

**Published:** 2025-09-05

**Authors:** I-Min Su, Yi-Yen Teng, Jer-Chuan Li, Chin-Hung Liu, Du-An Wu, Bang-Gee Hsu

**Affiliations:** 1 Department of Anesthesiology, Dalin Tzu Chi Hospital, Buddhist Tzu Chi Medical Foundation, Chiayi 62247, Taiwan; 2 School of Medicine, Tzu Chi University, Hualien 97004, Taiwan; 3 Institute of Medical Sciences, Tzu Chi University, Hualien 97004, Taiwan; 4 Department of Internal Medicine, Hualien Tzu Chi Hospital, Buddhist Tzu Chi Medical Foundation, Hualien 97004, Taiwan; 5 Division of Metabolism and Endocrinology, Buddhist Tzu Chi General Hospital, Hualien 97004, Taiwan; 6 Graduate Institute of Clinical Pharmacy, School of Medicine, Tzu Chi University, Hualien 97004, Taiwan; 7 School of Pharmacy, Tzu Chi University, Hualien 97004, Taiwan; 8 Division of Nephrology, Hualien Tzu Chi Hospital, Buddhist Tzu Chi Medical Foundation, Hualien 97004, Taiwan

**Keywords:** aortic stiffness, carotid–femoral pulse wave velocity, indoxyl sulfate, type 2 diabetes mellitus

## Abstract

*Background and Objectives*: Indoxyl sulfate (IS), a gut microbiota-derived metabolite of tryptophan, is implicated in vascular dysfunction through oxidative stress and inflammation. This study evaluated the association between serum IS levels and aortic stiffness (AS) in patients with type 2 diabetes mellitus (T2DM). *Materials and Methods*: This cross-sectional study enrolled 80 patients with T2DM from a medical center in eastern Taiwan, excluding patients with malignancy, acute infection, heart failure, or recent cardiovascular events. Serum IS concentrations were quantified using liquid chromatography–mass spectrometry. AS was assessed using carotid–femoral pulse wave velocity (cfPWV), with values > 10 m/s indicating AS. *Results*: A total of 30 patients (37.5%) had AS. IS levels were significantly higher in the AS group than in the control group (*p* < 0.001). After multivariate adjustment, IS remained an independent predictor of AS (odds ratio: 2.565, 95% confidence interval: 1.145–5.748, *p* = 0.022). Linear regression analysis confirmed IS as an independent contributor to cfPWV values (β = 0.261, *p* = 0.019). Receiver operating characteristic analysis showed fair discriminatory ability (area under the curve = 0.739, *p* < 0.001). *Conclusions*: In patients with T2DM, serum IS is an independent predictor of AS and may serve as a promising nontraditional biomarker for cardiovascular risk stratification.

## 1. Introduction

Globally, diabetes mellitus is one of the leading chronic diseases in terms of prevalence and clinical impact, contributing to severe disability, reduced life expectancy, and a rising economic burden [1]. In 2021, approximately 529 million individuals worldwide were living with diabetes mellitus, corresponding to a worldwide, age-adjusted prevalence of 6.1%. By 2050, it is estimated that 89 of the 204 countries and territories will have an age-standardized prevalence of diabetes mellitus greater than 10% [2]. In diabetes mellitus, cardiovascular disease (CVD) persists as the foremost factor driving morbidity and mortality [3]. Common cardiovascular (CV) complications of diabetes mellitus include peripheral arterial disease, stroke, heart failure, ischemic heart disease and coronary artery disease [4]. CVD pathogenesis in diabetes mellitus involves complex genetic, epigenetic, and cellular signaling disruptions that converge on metabolic and inflammatory pathways [5]. These systemic and molecular abnormalities cause structural and functional vascular changes [6], which are key prognostic markers in diabetes mellitus [7].

Arterial stiffness (AS), defined as reduced arterial elasticity or increased vascular rigidity, is a well-established CVD risk factor, with elevated fasting blood glucose as a known contributor [8]. Among arterial segments, aortic stiffness (AS), which reflects central arterial function, has strong prognostic significance. It correlates with diabetes mellitus duration and predicts future CV events [9]. Among noninvasive tools, carotid–femoral pulse wave velocity (cfPWV) is the reference standard for assessing AS. It measures the speed of the pressure wave between the carotid and femoral arteries and is inversely related to arterial compliance. Higher cfPWV indicates increased AS and is linked to adverse CV outcomes [10].

Indoxyl sulfate (IS), a gut microbiota-derived metabolite of tryptophan, is absorbed as indole and then converted in the liver before entering circulation. Although the kidney is the primary route of IS excretion, the gut–kidney axis plays a crucial role in regulating IS accumulation and its systemic effects [11]. Evidence shows IS contributes to vascular injury and arterial stiffening via inflammation, oxidative stress, renin–angiotensin–aldosterone system activation, and vascular calcification [12]. These mechanisms are relevant in diabetes mellitus, where early vascular dysfunction and impaired uremic toxin clearance may occur before overt nephropathy [13,14]. Although several studies have linked elevated IS levels to AS in chronic kidney disease (CKD) [15] and coronary artery disease [16], data on diabetes mellitus remain limited. As diabetes mellitus independently increases vascular dysfunction risk and often coexists with early renal impairment, clarifying IS’s role in aortic stiffening is essential. Therefore, we aimed to investigate the association between serum IS concentrations and AS, as measured by cfPWV, in patients with type 2 diabetes mellitus (T2DM).

## 2. Materials and Methods

### 2.1. Patients

This cross-sectional study enrolled 80 patients with confirmed T2DM between January and April 2020 at a medical center in eastern Taiwan. Approval for this study was granted by the Research Ethics Committee of Hualien Tzu Chi Hospital, Buddhist Tzu Chi Medical Foundation (IRB No. 110-139-C). All participants provided written informed consent prior to enrollment. Blood pressure (BP) was assessed in the seated position following a minimum of 10 min of rest. Systolic (SBP) and diastolic (DBP) blood pressures were recorded in the morning using a standardized protocol. Patients with a history of malignancy, acute infection, heart failure, limb amputation, acute myocardial infarction, or acute coronary syndrome, and those who declined to give consent were excluded. Hypertension and diabetes mellitus were diagnosed using clinical criteria: SBP equal to or greater than 140 mmHg, DBP equal to or greater than 90 mmHg, fasting plasma glucose equal to or greater than 126 mg/dL, or current use of antihypertensive or antidiabetic medication.

### 2.2. Anthropometric Analysis and Biochemical Determinations

Waist circumference was assessed with a nonelastic tape at the midpoint between the lowest rib and the iliac crest. Participants’ body weight and height were obtained while wearing light clothing and barefoot. Body mass index was derived as weight (kg) divided by height squared (m^2^). Fasting venous blood (~5 mL) was collected and centrifuged at 3000× *g* for 10 min. Serum levels of fasting glucose, HbA1c, blood urea nitrogen (BUN), creatinine, triglycerides, total cholesterol, LDL-C, HDL-C, and C-reactive protein (CRP) were measured using an automated analyzer (Siemens Advia 1800; Siemens Healthcare GmbH, Erlangen, Germany). The urine albumin-to-creatinine ratio (UACR) was obtained from spot urine samples, while the estimated glomerular filtration rate (eGFR) was determined using the Chronic Kidney Disease Epidemiology Collaboration equation.

### 2.3. Carotid–Femoral Pulse Wave Velocity Assessment

AS was assessed via cfPWV using applanation tonometry (SphygmoCor system, AtCor Medical, Sydney, NSW, Australia). All assessments took place in a temperature-controlled, quiet room, with patients in the supine position following a minimum of 10 min of rest. Pulse waveforms were recorded sequentially at the carotid and femoral arteries, along with electrocardiogram signals. Transit time was averaged over 10 cardiac cycles using the R-wave as a reference. Carotid–femoral distance was derived by subtracting the distance from the carotid artery to the sternal notch from that between the sternal notch and the femoral site. cfPWV was determined by dividing distance by transit time. Quality control indices ensured data consistency. AS was considered present when cfPWV was greater than 10 m/s, based on the 2018 ESC guidelines for cardiovascular risk [17].

### 2.4. High-Performance Liquid Chromatography–Mass Spectrometry for IS Measurements

A validated liquid chromatography–mass spectrometry method, based on prior study [16], was employed to measure total serum IS levels. A mixture was prepared by combining 100 µL of serum with 100 µL of 50 mM sodium phosphate dibasic heptahydrate and spiked with a stable isotope-labeled internal standard (d4-IS). Extraction was performed using Novum^®^ Simplified Liquid Extraction cartridges (Phenomenex, Torrance, CA, USA). After evaporation under nitrogen, the residue was reconstituted in 100 µL of methanol. Separation was conducted on a Waters^®^ e2695 HPLC system (Waters Corporation, Milford, MA, USA) with an ACQUITY QDa^®^ mass detector and a Luna^®^ C18(2) column (5 µm, 250 × 4.6 mm, 100 Å) (Phenomenex, Torrance, CA, USA) at 40 °C. The mobile phase was prepared using 0.1% formic acid in double-distilled water as component A and methanol as component B, with a flow rate of 0.8 mL/min. Injection volume was 30 µL. A linear gradient from 5% to 70% B over 14 min was applied, held at 70% B for 2 min, and returned to 50% B. Mass detection used negative-ion electrospray ionization at 400 °C, with a capillary voltage of 0.8 kV and a sample cone voltage of 15.0 V. Single-ion recording mode detected *m*/*z* 211.9 for IS and 216.0 for d4-IS. Empower^®^ version 3.0 was applied for recording and analyzing the data. IS levels were calculated using a calibration curve from known standards.

### 2.5. Statistical Analysis

All statistical analyses were conducted with SPSS software, version 19.0 (IBM Corp., Armonk, NY, USA). The Kolmogorov–Smirnov test was applied to examine data normality. Normally distributed variables were presented as mean ± standard deviation and compared using the independent samples *t*-test. Non-normally distributed variables were shown as medians with interquartile ranges and compared using the Mann–Whitney U test. Categorical variables were analyzed using the chi-square test and expressed as frequencies (in percentages). Logarithmic transformation (base-10) was applied to skewed variables for parametric analysis. Simple linear regression assessed correlations between clinical variables and cfPWV. Variables significantly associated with cfPWV were included in a multivariate forward stepwise regression. Multivariate logistic regression identified independent predictors of AS. Receiver operating characteristic (ROC) curve analysis evaluated the ability of serum IS levels to predict AS, and the area under the curve (AUC) was calculated (MedCalc Software Ltd., version 22.019, Ostend, Belgium). A two-tailed *p*-value <0.05 was considered statistically significant.

## 3. Results

### 3.1. Baseline Characteristics

In this cohort of 80 patients with type 2 diabetes mellitus, 30 (37.5%) were identified as having AS (cfPWV > 10 m/s), while the remaining 50 (62.5%) served as controls (Table 1). Compared with controls, patients with AS were significantly older (*p* = 0.007) and exhibited higher SBP (*p* = 0.002), but not DBP (*p* = 0.186). Metabolic disturbances were more pronounced in the AS group, as reflected by elevated triglycerides (*p* = 0.023), fasting glucose (*p* = 0.025), and HbA1c (*p* = 0.013) levels. Renal function was also compromised, with lower eGFR (*p* = 0.003) accompanied by higher serum creatinine (*p* = 0.047), BUN (*p* = 0.013), and UACR (*p* = 0.005). In addition, systemic inflammation and uremic toxin burden appeared greater among these patients, evidenced by elevated CRP (*p* = 0.029) and markedly higher serum IS (*p* < 0.001) concentrations. Hypertension (*p* = 0.037) was more frequently observed in the AS group, whereas anthropometric measures, broader lipid profiles, and medication use did not differ significantly between groups.

### 3.2. Serum IS Level and Development of AS

**Figure 1:**
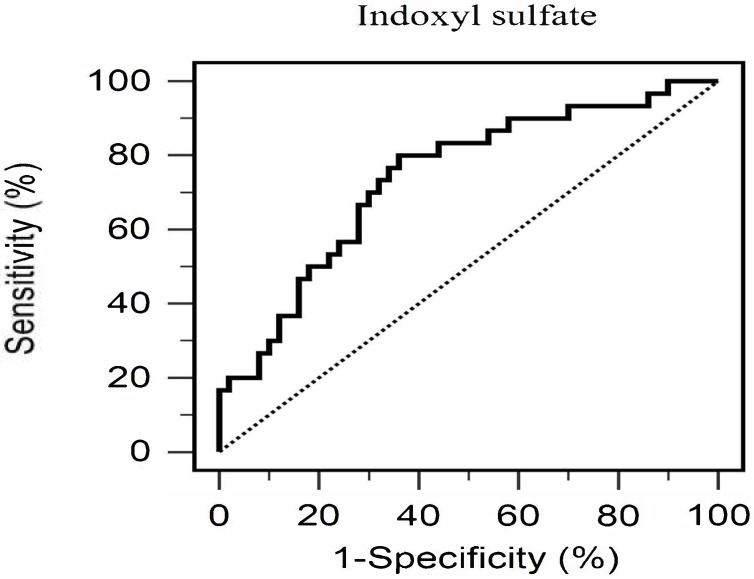
Diagnostic Performance of Serum Total Indoxyl Sulfate for Predicting Aortic Stiffness in Patients with Type 2 Diabetes Mellitus (*n* = 80).

Multivariate logistic regression was conducted to identify independent predictors of AS, incorporating variables that showed significant differences in univariate analyses, namely age, hypertension, SBP, triglycerides, fasting glucose, HbA1c, BUN, serum creatinine, eGFR, UACR, CRP, and serum IS levels (Table 2). After adjustment for these covariates, serum IS remained the only independent predictor of AS (odds ratio: 2.565, 95% confidence interval [CI]: 1.145–5.748, *p* = 0.022), indicating that each 1 μg/mL increase in serum IS was associated with a 2.565-fold higher likelihood of AS. The diagnostic utility of serum IS was further assessed by ROC curve analysis, which demonstrated fair discriminative ability with an AUC of 0.739 (95% CI: 0.629–0.831, *p* < 0.001) (Figure 1). The optimal cutoff value determined by the Youden index was 0.796 μg/mL, yielding a sensitivity of 80.0% and a specificity of 64.0%. At this threshold, the positive predictive value was 57.14% and the negative predictive value was 84.21%, underscoring the clinical potential of serum IS as a biomarker for identifying AS in patients with T2DM.

### 3.3. Correlations Between cfPWV Levels and Clinical Variables

Simple linear regression analysis was conducted to explore clinical determinants of cfPWV, as summarized in Table 3. Significant positive correlations were observed between cfPWV and age (*r* = 0.289, *p* = 0.009), SBP (*r* = 0.358, *p* = 0.001), log-transformed triglycerides (log-triglycerides, *r* = 0.239, *p* = 0.033), log-glucose (*r* = 0.234, *p* = 0.037), log-UACR (*r* = 0.272, *p* = 0.014), log-CRP (*r* = 0.292, *p* = 0.008), and serum IS (*r* = 0.351, *p* = 0.001), whereas eGFR demonstrated a significant negative correlation (*r* = −0.302, *p* = 0.006). In multivariate forward stepwise linear regression, SBP (β = 0.272, adjusted R^2^ change = 0.117, *p* = 0.015) and serum IS (β = 0.261, adjusted R^2^ change = 0.051, *p* = 0.019) emerged as independent predictors of cfPWV. Notably, the contribution of serum IS to AS was independent of most conventional CV risk factors and exhibited an effect size comparable to that of SBP, underscoring its potential role as a novel determinant of vascular dysfunction in patients with T2DM.

### 3.4. Spearman Correlation Analysis of Serum IS Level and Clinical Variables

Spearman correlation analysis was performed to examine the associations between serum IS levels and clinical variables (Table 4). Higher serum IS concentrations were significantly correlated with older age (*r* = 0.231, *p* = 0.039), elevated SBP (*r* = 0.331, *p* = 0.003), increased cfPWV (*r* = 0.351, *p* = 0.001), higher log-glucose (*r* = 0.224, *p* = 0.046), log-creatinine (*r* = 0.229, *p* = 0.041), and log-CRP (*r* = 0.338, *p* = 0.002). Conversely, IS levels were negatively correlated with eGFR (*r* = −0.286, *p* = 0.010). Collectively, these findings demonstrate that a greater IS burden is closely linked with CV, metabolic, inflammatory, and renal dysfunction in patients with T2DM, reinforcing its potential role as an integrative marker of systemic vascular risk.

## 4. Discussion

This cross-sectional study provides evidence that serum IS concentrations independently predict AS in patients with T2DM, expanding our understanding of nontraditional CV risk factors in this high-risk population. The key finding that IS remains significantly associated with AS after adjusting for established CV risk factors suggests that IS contributes to diabetic vascular complications through distinct pathophysiological mechanisms.

AS is a core feature of vascular aging and a strong predictor of CV outcomes in diabetes mellitus [18]. Its pathogenesis involves complex molecular and cellular processes, including vascular smooth muscle cell (VSMC) phenotypic switching, extracellular matrix remodeling, and progressive arterial wall calcification [19]. Although aging, hyperglycemia, hypertension, and dyslipidemia are known contributors to arterial stiffening in diabetes mellitus [20], our findings highlight the additional role of IS. The independent predictive value of IS in our multivariate analysis suggests that conventional CV risk assessment may be incomplete in patients with T2DM. This aligns with growing recognition that diabetes mellitus-related vascular dysfunction extends beyond glycemic control and involves broader metabolic and toxin-mediated pathways [21,22]. The persistent association between IS and AS, even after controlling for SBP, renal function, and inflammatory markers, indicates that IS acts through mechanisms distinct from hemodynamic stress and traditional CV risks.

In our cohort, participants with AS were significantly older and had high levels of SBP, fasting glucose, HbA1c, triglycerides, and more impaired renal function, indicated by elevated BUN and serum creatinine, lower eGFR, and increased levels of CRP and UACR. These findings reaffirm the roles of metabolic dysregulation, inflammation, and renal dysfunction in AS pathogenesis [23]. However, after multivariate adjustment, only serum IS remained an independent predictor of AS. This suggests that, although traditional cardiometabolic factors are associated with AS, their predictive value may be eclipsed by nontraditional toxins like IS, highlighting the need to broaden risk assessment in T2DM.

IS, a tryptophan-derived metabolite, exerts several vascular effects that may promote AS [11]. Emerging evidence suggests that IS levels can be elevated in patients with T2DM even when eGFR is preserved. This elevation may reflect early tubular dysfunction rather than glomerular decline and is potentially driven by T2DM-related gut microbiota dysbiosis and altered tryptophan metabolism [24,25]. It induces VSMC proliferation and osteogenic differentiation, leading to medial calcification [26] and increased pulse wave velocity [27]. IS also causes endothelial dysfunction via oxidative stress, reduced nitric oxide bioavailability, and inflammatory pathway activation [28,29]. These effects create a proatherogenic environment that promotes arterial stiffening. The fact that IS can be elevated before overt kidney impairment highlights its potential role as an early mediator of vascular dysfunction in T2DM, independent of traditional risk factors and renal function. The observed positive correlation between IS and inflammatory markers supports this mechanism. Additionally, the association between IS and renal dysfunction highlights the importance of the kidney–heart axis in diabetic complications [30].

In patients with T2DM, who are prone to early vascular dysfunction and impaired uremic toxin clearance even before overt nephropathy [13], these mechanisms may be especially relevant. Linear regression identified SBP and serum IS as independent determinants of cfPWV. The retention of IS in the final model, despite adjustment for age, hypertension, and renal function, underscores its independent role. This supports the hypothesis that IS directly contributes to arterial remodeling and stiffening through VSMC calcification, oxidative stress, and endothelial dysfunction [11,12,26].

Spearman correlation analysis further supports IS’s pathogenic role, showing significant associations with SBP, cfPWV, fasting glucose, serum creatinine, CRP, and age and a negative correlation with eGFR. These findings suggest that IS serves as an integrated marker of metabolic, renal, and vascular stress. The positive correlation with CRP reinforces inflammation’s role in IS-mediated vascular injury, whereas the inverse correlation with eGFR highlights the renal–vascular axis as a key factor in AS development among patients with T2DM.

ROC curve analysis yielded an AUC of 0.739, demonstrating fair discriminatory power of serum IS for predicting AS. The identified cutoff of 0.796 μg/mL showed high sensitivity (80%) and moderate specificity (64%), suggesting that IS could serve as a useful screening biomarker for vascular risk in T2DM. The liquid chromatography–mass spectrometry assay is increasingly accessible and cost-effective, making it suitable for routine laboratory panels, especially in primary care settings lacking advanced CV imaging. Unlike traditional risk factors, IS is modifiable through dietary and microbiome-targeted interventions. Early identification of elevated IS may enable preventive strategies beyond conventional CV risk management [31].

Our findings align with previous studies showing associations between IS and AS in CKD and coronary artery disease populations [16]. However, this study extends those observations to individuals with T2DM, addressing a key knowledge gap. The strength of the association suggests that patients with T2DM may be particularly vulnerable to IS-mediated vascular injury, possibly due to metabolic abnormalities that amplify toxin-related pathways. The correlations between IS and age, BP, and renal function markers are consistent with earlier research [32,33], further supporting the validity of our results. Importantly, the independent predictive value of IS for AS in T2DM is a novel contribution, highlighting the clinical relevance of IS in this population. By integrating conventional CV risk factors with emerging nontraditional biomarkers such as IS, our findings support a more comprehensive approach to vascular risk stratification in T2DM. Future longitudinal and interventional studies are required to confirm IS as a therapeutic target and to determine whether IS-lowering strategies can reduce vascular stiffness and CV events in this high-risk group.

Several limitations of the present study warrant consideration. First, the cross-sectional design limits causal inference regarding the IS–AS relationship. Longitudinal studies are needed to determine whether elevated IS precedes or results from AS. Second, we measured total IS rather than free IS, which may be more biologically active. Third, the modest sample size may limit generalizability to broader diabetic populations. Although the initial power analysis indicated that 85 participants were required to detect a moderate correlation (*r* = 0.30) at α of 0.05 and power of 80% [34], the observed correlation between serum IS and cfPWV was *r* = 0.351. A post hoc power analysis using this effect size and a sample size of 80 indicated a power of approximately 90%, suggesting sufficient statistical power to detect the observed association. Fourth, we did not assess dietary tryptophan intake or gut microbiota composition, which may influence IS levels. Finally, this study was conducted in a single center in eastern Taiwan, which may limit external validity to other populations.

## 5. Conclusions

In conclusion, this study shows that serum IS concentrations are independently associated with AS in patients with T2DM, underscoring the role of IS in diabetic vascular complications. These findings may guide biomarker development and inform therapies to reduce CV risk in this high-risk group. The results emphasize the need to consider nontraditional risk factors beyond standard CV assessments and support the potential clinical utility of IS measurement for improved risk stratification.

## Figures and Tables

**Table 1:** Clinical variables of the 80 patients with type 2 diabetes mellitus with control (cfPWV ≤ 10.0 m/s) or aortic stiffness group (cfPWV > 10.0 m/s).

Characteristic	All Patients (*n* = 80)	Control Group (*n* = 50)	Aortic Stiffness Group (*n* = 30)	*p* Value
Age (years)	61.84 ± 13.09	58.85 ± 13.15	66.87 ± 11.52	0.007 *
Height (cm)	161.78 ± 8.35	163.06 ± 8.32	159.63 ± 8.09	0.075
Body weight (kg)	70.45 ± 14.79	70.69 ± 16.12	70.06 ± 12.51	0.856
Body mass index (kg/m^2^)	26.79 ± 4.36	26.42 ± 4.64	27.39 ± 3.85	0.339
cfPWV (m/s)	9.49 ± 2.90	7.72 ± 1.39	12.45 ± 2.29	<0.001 *
SBP (mmHg)	142.65 ± 21.03	137.10 ± 19.60	151.90 ± 20.34	0.002 *
DBP (mmHg)	81.96 ± 12.36	80.54 ± 12.58	84.33 ± 11.81	0.186
Total cholesterol (mg/dL)	162.41 ± 31.26	158.12 ± 27.77	169.57 ± 35.69	0.113
Triglyceride (mg/dL)	122.50 (85.00–170.50)	104.00 (72.00–159.50)	133.00 (103.25–189.00)	0.023 *
HDL-C (mg/dL)	47.11 ± 12.14	46.68 ± 12.19	47.83 ± 12.23	0.683
LDL-C (mg/dL)	98.31 ± 26.14	97.94 ± 23.18	98.93 ± 30.86	0.871
Fasting glucose (mg/dL)	136.00 (123.50–176.00)	130.00 (122.50–158.00)	161.50 (124.75–210.75)	0.025 *
HbA1c (%)	7.50 (6.73–8.98)	7.00 (6.60–8.25)	8.10 (7.00–9.50)	0.013 *
Blood urea nitrogen (mg/dL)	16.00 (12.25–20.00)	15.00 (12.00–17.25)	18.00 (14.00–22.75)	0.013 *
Creatinine (mg/dL)	0.90 (0.70–1.08)	0.80 (0.70–1.00)	0.90 (0.80–1.20)	0.047 *
eGFR (mL/min)	84.13 ± 28.59	91.24 ± 29.28	72.27 ± 23.34	0.003 *
UACR (mg/g)	19.19 (7.82–114.73)	11.50 (7.35–45.13)	37.87 (12.62–348.18)	0.005 *
C-reactive protein (mg/dL)	0.13 (0.07–0.41)	0.11 (0.07–0.22)	0.25 (0.09–0.59)	0.029 *
Indoxyl sulfate (μg/mL)	1.05 ± 0.78	0.81 ± 0.60	1.46 ± 0.89	<0.001 *
Male, *n* (%)	42 (52.5)	29 (58.0)	13 (43.3)	0.203
Hypertension, *n* (%)	44 (55.0)	23 (46.0)	21 (70.0)	0.037 *
ACE inhibitor use, *n* (%)	7 (8.8)	3 (6.0)	4 (13.3)	0.261
ARB use, *n* (%)	31 (38.8)	18 (36.0)	13 (43.3)	0.515
β-blocker use, *n* (%)	10 (12.5)	5 (10.0)	5 (16.7)	0.383
CCB use, *n* (%)	25 (31.3)	13 (26.0)	12 (40.0)	0.191
Statin use, *n* (%)	46 (57.5)	26 (52.0)	20 (66.7)	0.199
Fibrate use, *n* (%)	10 (12.5)	7 (14.0)	3 (10.0)	0.600
Metformin use, *n* (%)	41 (51.3)	23 (46.0)	18 (60.0)	0.225
Sulfonylureas use, *n* (%)	42 (52.5)	27 (54.0)	15 (50.0)	0.729
DDP-4 inhibitor use, *n* (%)	46 (57.5)	31 (62.0)	15 (50.0)	0.293
Insulin use, *n* (%)	22 (27.5)	13 (26.0)	9 (30.0)	0.698

Data for continuous variables are reported as mean ± standard deviation and were assessed using Student’s *t*-test. Non-normally distributed variables are summarized as median with interquartile range and analyzed via the Mann–Whitney U test. Categorical data are expressed as number and percentage, with group comparisons performed using the chi-square test. The following abbreviations are used: cfPWV (carotid–femoral pulse wave velocity), SBP (systolic blood pressure), DBP (diastolic blood pressure), HDL-C (high-density lipoprotein cholesterol), LDL-C (low-density lipoprotein cholesterol), HbA1c (glycated hemoglobin), eGFR (estimated glomerular filtration rate), UACR (urine albumin-to-creatinine ratio), ACE (angiotensin-converting enzyme), ARB (angiotensin receptor blocker), CCB (calcium channel blocker), and DDP-4 (dipeptidyl peptidase 4). * Statistical significance was set at *p* < 0.05.

**Table 2:** Factors Associated with Aortic Stiffness in Patients with Type 2 Diabetes Mellitus: Multivariate Logistic Regression Analysis (*n* = 80).

Variables	Odds Ratio	95% Confidence Interval	*p* Value
Indoxyl sulfate, 1 μg/mL	2.565	1.145–5.748	0.022 *
Age, 1 year	1.042	0.977–1.111	0.213
Hypertension, present	0.538	0.088–3.304	0.503
Systolic blood pressure, 1 mmHg	1.041	0.987–1.099	0.141
C-reactive protein, 1 mg/dL	0.598	0.099–3.626	0.576
Triglyceride, 1 mg/dL	1.003	0.994–1.013	0.482
Fasting glucose, 1 mg/dL	1.008	0.988–1.028	0.445
HbA1c, 1%	0.948	0.533–1.686	0.855
Blood urea nitrogen, 1 mg/dL	1.039	0.912–1.184	0.562
Creatinine, 0.1 mg/dL	0.915	0.601–1.394	0.681
Estimated glomerular filtration rate, 1 mL/min	0.991	0.936–1.049	0.755
Urine albumin-to-creatinine ratio, 1 mg/g	1.000	0.999–1.001	0.980

Data analysis was done using the multivariate logistic regression analysis (adopted factors: age, hypertension, systolic blood pressure, C-reactive protein, triglyceride, fasting glucose, HbA1c, blood urea nitrogen, creatinine, estimated glomerular filtration rate, urine albumin-to-creatinine ratio, and indoxyl sulfate). HbA1c, glycated hemoglobin. * *p* < 0.05 was considered statistically significant.

**Table 3:** Association of Carotid–Femoral Pulse Wave Velocity with Clinical Variables in 80 Patients with Type 2 Diabetes Mellitus.

Clinical Variables	Carotid–Femoral Pulse Wave Velocity (m/s)				
	Univariate Regression		Multivariate Regression		
	*r*	*p* Value	β	Adjusted R^2^ Change	*p* Value
Age (years)	0.289	0.009 *	—	—	—
Height (cm)	−0.204	0.069	—	—	—
Body weight (kg)	0.023	0.839	—	—	—
Body mass index (kg/m^2^)	0.163	0.149	—	—	—
SBP (mmHg)	0.358	0.001 *	0.272	0.117	0.015 *
DBP (mmHg)	0.214	0.057	—	—	—
Total cholesterol (mg/dL)	0.163	0.149	—	—	—
Log-Triglyceride (mg/dL)	0.239	0.033 *	—	—	—
HDL-C (mg/dL)	0.066	0.559	—	—	—
LDL-C (mg/dL)	0.138	0.222	—	—	—
Log-Glucose (mg/dL)	0.234	0.037 *	—	—	—
Log-HbA1c (%)	0.148	0.191	—	—	—
Log-BUN (mg/dL)	0.218	0.052	—	—	—
Log-Creatinine (mg/dL)	0.219	0.051	—	—	—
eGFR (mL/min)	−0.302	0.006 *	—	—	—
Log-UACR (mg/g)	0.272	0.014 *	—	—	—
Log-CRP (mg/dL)	0.292	0.008 *	—	—	—
Indoxyl sulfate (μg/mL)	0.351	0.001 *	0.261	0.051	0.019 *

Data for triglycerides, glucose, HbA1c, BUN, creatinine, UACR, and CRP showed skewed distributions and were log-transformed before analysis. Associations were evaluated using univariate linear regression, followed by multivariate stepwise linear regression adjusting for hypertension, age, SBP, log-triglycerides, log-glucose, eGFR, log-UACR, log-CRP, and indoxyl sulfate. The following abbreviations are used: systolic blood pressure (SBP); diastolic blood pressure (DBP); high-density lipoprotein cholesterol (HDL-C); low-density lipoprotein cholesterol (LDL-C); glycated hemoglobin (HbA1c); blood urea nitrogen (BUN); estimated glomerular filtration rate (eGFR); urine albumin-to-creatinine ratio (UACR); C-reactive protein (CRP). * Statistical significance was set at *p* < 0.05.

**Table 4:** Association Between Serum Total Indoxyl Sulfate Levels and Clinical Variables.

Variables	Spearman’s Correlation Coefficient	*p* Value
Age (years)	0.231	0.039 *
Body mass index (kg/m^2^)	0.046	0.686
SBP (mmHg)	0.331	0.003 *
DBP (mmHg)	0.075	0.511
Carotid–femoral PWV (m/s)	0.351	0.001 *
Total cholesterol (mg/dL)	0.003	0.981
Log-Triglyceride (mg/dL)	0.118	0.295
HDL-C (mg/dL)	0.033	0.768
LDL-C (mg/dL)	−0.029	0.795
Log-Glucose (mg/dL)	0.224	0.046 *
Log-HbA1c (%)	0.204	0.069
Log-BUN (mg/dL)	0.203	0.072
Log-Creatinine (mg/dL)	0.229	0.041 *
eGFR (mL/min)	−0.286	0.010 *
Log-UACR (mg/g)	0.215	0.055
Log-CRP (mg/dL)	0.338	0.002 *

Data for triglycerides, glucose, HbA1c, BUN, creatinine, UACR, and CRP showed skewed distributions and were log-transformed before analysis. The following abbreviations are used: systolic blood pressure (SBP); diastolic blood pressure (DBP); pulse wave velocity (PWV); high-density lipoprotein cholesterol (HDL-C); low-density lipoprotein cholesterol (LDL-C); glycated hemoglobin (HbA1c); blood urea nitrogen (BUN); estimated glomerular filtration rate (eGFR); urine albumin-to-creatinine ratio (UACR); C-reactive protein (CRP). * Statistical significance was set at *p* < 0.05 (2-tailed).

## Data Availability

Upon request, the corresponding author can provide the data utilized in this study.

## Abbreviations

The following abbreviations are used in this manuscript:

AS	aortic stiffness
AUC	area under the curve
BP	blood pressure
BUN	blood urea nitrogen
CV	cardiovascular
CVD	cardiovascular disease
cfPWV	carotid–femoral pulse wave velocity
CKD	chronic kidney disease
CRP	C-reactive protein
DBP	diastolic blood pressure
eGFR	estimated glomerular filtration rate
HbA1c	glycated hemoglobin
IS	indoxyl sulfate
ROC	receiver operating characteristic
SBP	systolic blood pressure
T2DM	type 2 diabetes mellitus
UACR	urine albumin-to-creatinine ratio
